# Carcinoembryonic Antigen (CEA): Origin, Role in Oncology, and Concentrations in Serum and Peritoneal Fluid

**DOI:** 10.3390/jcm14093189

**Published:** 2025-05-05

**Authors:** Julia Niedzielska, Tomasz Jastrzębski

**Affiliations:** 1 Department of Gynecology, Obstetrics and Neonatology, Medical University of Gdańsk, 80-214 Gdańsk, Poland; tomasz.jastrzebski@gumed.edu.pl; 2 Department of Oncological Surgery, PCZ Brzeziny Hospital, 95-060 Brzeziny, Poland

**Keywords:** CEA, cancer, serum, ascites, peritoneal fluid

## Abstract

CEA (carcinoembryonic antigen), which belongs to the acidic glycoproteins, is primarily produced during the fetal period. Following this stage, low levels of CEA are considered physiological, while elevated concentrations are associated with a range of both benign and malignant pathologies. The liver plays a key role in CEA metabolism. The most common material used to determine CEA concentrations by various techniques is blood, and measuring CEA in peritoneal fluid holds clinical value. CEA has been found to contribute to carcinogenesis, metastasis, and treatment resistance. Therefore, its serum concentration is widely used in oncology for prognosis, disease monitoring, and recurrence detection, despite its limited sensitivity and specificity, which prevent it from serving as a standalone diagnostic tool. Elevated serum CEA levels are linked to worse outcomes in lung, liver, breast, colorectal, and pancreatic cancers. Imaging and multi-marker panels that include CEA enhance diagnostic accuracy, but its role remains context-dependent and varies by cancer type. CEA levels in peritoneal fluid have been explored as a potential marker for detecting malignancy and predicting recurrence, particularly in gastric, gynecological, and colorectal cancers. Peritoneal fluid CEA has also been proven useful in differentiating the etiology of ascites. While cytology remains the standard for the detection of tumor cells in body fluids, its limited sensitivity provides a strong rationale for incorporating peritoneal fluid CEA measurements as a complementary diagnostic tool, potentially alongside other markers. Additionally, the lack of standardized measurement techniques and cut-off values underlines the methodological challenges that still need to be addressed in future research for both serum and peritoneal CEA levels.

## 1. Introduction

CEA (carcinoembryonic antigen) is a glycoprotein produced during the fetal period and it is present only in small amounts afterwards. Under normal conditions, CEA is produced in the pancreas, intestines, and liver, and the upper limit of its concentration should not exceed 5 ng/mL [1,2]. It is also a non-specific tumor marker derived from tumor cells [3,4], meaning that it can reflect a pathological process but is not uniquely associated with a particular disease or condition. Therefore, as useful as CEA may be, its interpretation must be placed within a broader clinical context and supported by further investigation.

The CEA molecule belongs to the acidic glycoproteins and presents a significant degree of variability, both at the intermolecular and intramolecular level [4,5]. The varying sialylation of this molecule is likely one of the factors underlying its biochemical heterogeneity [6]. Detection of CEA in both membrane-associated (hydrophobic) and soluble (hydrophilic) forms indicates that its transition from a cell-bound to a freely circulating state may depend on alterations in its molecular structure [7]. The exact process of the spontaneous release of CEA into the serum from normal and cancerous cells most likely arises from different mechanisms and has yet to be fully elucidated. To date, one of the studies showed that an endogenous glycosylphosphatidylinositol-specific phospholipase D (GPI-PLD) may play a role in the spontaneous release of CEA from human colon carcinoma cells [8]. Another article reported that phosphatidylinositol-specific phospholipase C plays a key role in releasing CEA from the cell membrane and converting it into its soluble, hydrophilic form in a human colonic adenocarcinoma cell line [9]. In contrast, it has also been demonstrated that a non-proteolytic cleavage can be responsible for shedding CEA from normal colonic epithelial cells [10]. The catabolism of CEA is probably mediated by Kupffer cells, which transport the molecule to the liver, where it undergoes degradation involving lysosomes [5]. More precisely, the CEA molecule present in the sinusoid passes through a Kupffer cell—by binding a peptide sequence in its N-terminal region to a specific Ca^2+^-dependent receptor—where a sialic acid residue is removed [7]. Subsequently, asialo-CEA is endocytosed by hepatocytes via the asialoglycoprotein receptor, forming a coated vesicle that fuses with a lysosome and is later degraded in the Golgi apparatus [7]. A study using rats demonstrated that over 80% of CEA is metabolized by the liver [11]. This substantial hepatic involvement in CEA metabolism renders the liver a pivotal factor in investigating the underlying causes of elevated CEA concentrations. It is noteworthy that, despite the presence of CEA receptors on alveolar macrophages, these cells metabolize CEA at a rate that is approximately fourfold slower than that observed in hepatocytes. This observation suggests that the role of alveolar macrophages in this process is relatively minor. To date, no CEA receptors have been detected on peritoneal macrophages [7].

It has been demonstrated that a proportion of the CEA metabolized by the liver subsequently enters the bile, thereby indicating that cholestasis-inducing conditions may also contribute to an increase in CEA levels in the blood [5,7].

Other causes of elevated CEA levels include a number of benign conditions, such as liver inflammation and fibrosis, pancreatitis, gastric and duodenal ulcers, non-cancerous lung diseases (including inflammation, fibrosis and chronic obstructive pulmonary disease), chronic renal failure, Alzheimer’s disease, and non-cancerous breast diseases [5,7,12]. An increase in CEA can also indicate the presence of malignant disease, such as liver, lung, pancreatic, breast, or colorectal cancer [5,7,12].

**Figure 1:**
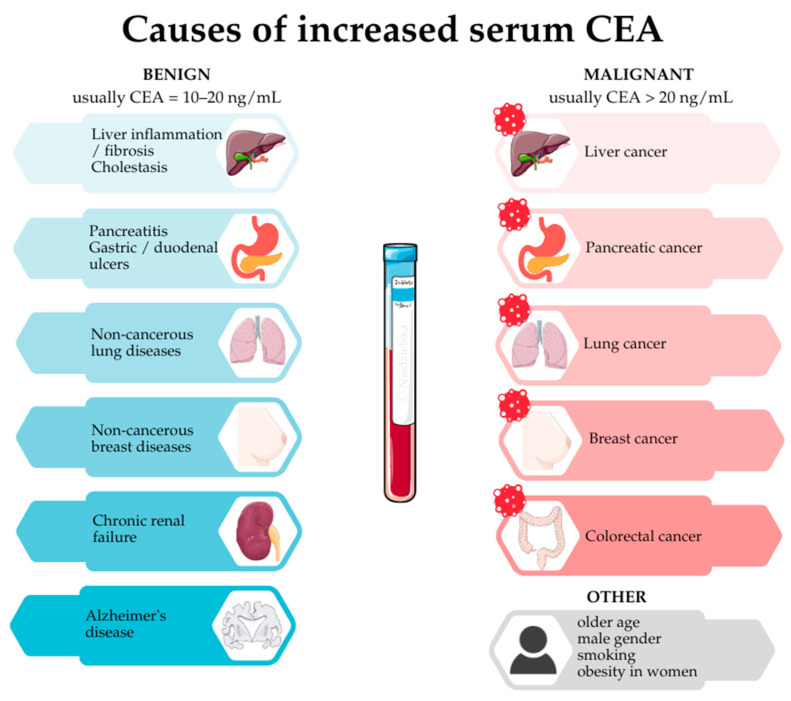
Causes of increased serum CEA levels.

In simple terms, it can be assumed that non-cancerous causes provoke a lower rise in serum CEA (in the range 10–20 ng/mL), whereas if cancer is the underlying cause, the values exceed 20 ng/mL, especially when dealing with disseminated disease [1,5]. However, it is not a rule that can be universally applied as there are patients with metastatic disease with low levels of CEA in the blood [13]. In addition, it is worth remembering that factors such as older age, male gender, smoking, and obesity in women may contribute to slightly elevated CEA levels [12,14]. As a result, not every deviation of CEA concentration from the norm indicates the presence of a serious pathology. Figure 1 provides a brief graphical summary of the causes of increased serum CEA levels.

CEA has been demonstrated to play a pivotal role in the formation and survival of cancer cells. In addition, it is known to contribute to carcinogenesis by disrupting cellular differentiation and promoting anti-apoptotic activity [7,15,16]. Furthermore, CEA has been shown to be involved in metastasis formation by inducing angiogenesis, migration, and adhesion of tumor cells [7,15,16]. Due to the phenomenon of CEA-induced activation of endothelial cells, colorectal cancer patients with elevated serum CEA levels exhibited increased tumor microvascularization [17]. The literature suggests that the shedding of CEA by tumor cells functions as an anti-adhesive adaptation, protecting malignant cells from lymphocyte-mediated attack [18]. Conversely, another study reported that downregulation of CEA reduced tumor cell aggregation by 70% in vivo [19]. These findings indicate that the adhesive properties of CEA may depend on its surface density and potentially vary across different phases of carcinogenesis [19]. Stimulation of Kupffer cells by CEA leads to the production of pro-inflammatory cytokines, such as IL-1β (interleukin 1 beta) and TNF-α (tumor necrosis factor-alpha) [20]. These cytokines are known to enhance the expression of adhesion molecules and promote tumor cell adhesion, which in turn facilitates metastasis. In addition, evidence suggests a potential link between CEA and chemotherapeutic resistance [16]. It has been established that CEA can act as a survival factor for colon cancer cells by downregulating apoptotic gene expression across diverse biological conditions, including treatment with 5-fluorouracil [21]. CEA molecules are present on the surface of tumor cells, most abundantly on their luminal surface, and are released into the bloodstream, metastatic foci, and the body fluids surrounding the tumor [4,5]. As a result of this release, CEA levels in various biological matrices—including serum, peritoneal fluid, and pleural fluid—have emerged as a valuable diagnostic tool for clinicians [22]. For instance, elevated CEA levels in pleural fluid (i.e., >5 ng/mL) have been shown to be an independent predictor of significantly worse overall survival (OS) in patients with non-small cell lung cancer (NSCLC) [23]. CEA can also be measured in body fluids such as saliva, urine, vaginal fluid, and semen [24,25,26,27]. However, its role in this context has not yet been extensively explored, and its clinical utility remains very limited according to current knowledge.

While some authors consider the concentration of CEA and other markers such as CA125 (cancer antigen 125) or CA19-9 (cancer antigen 19-9) as complementary diagnostics in oncology, others believe that since their serum and ascites concentrations are correlated, they do not add any clinical benefit [22,28]. The ambiguity surrounding this issue is further compounded by the heterogeneity of many studies in terms of the etiology of ascites, the cut-off points for CEA values, and the methodologies employed for its measurement.

## 2. Cancer—Serum CEA

For the aforementioned reasons, serum CEA concentration is a marker that is commonly utilized in clinical practice. However, it lacks sufficient specificity or sensitivity to serve as an independent basis for cancer diagnosis. Nevertheless, CEA provides valuable information regarding the likelihood of cancer presence, its stage, and the presence of metastases, in addition to facilitating the monitoring of the disease course and its potential recurrence [5,12]. Given the mentioned limitations of serum CEA levels in terms of sensitivity, it is recommended that additional diagnostic methods (including imaging) be employed in conjunction with CEA measurement to ensure the adequacy of patient monitoring [29].

With regard to lung cancer, a review, which included 23 studies, concluded that elevated blood CEA levels in patients with NSCLC are associated with an increased risk of relapse and mortality, irrespective of the treatment modality employed [30]. It is worth noting that not only single-point measurements, but also serial assessments and the dynamics of serum CEA concentration have clinical relevance. A retrospective study involving 2959 patients undergoing surgery for stage I–III NSCLC analyzed serum CEA levels measured using chemiluminescence immunoassay from the preoperative period up to three years post-surgery [31]. Patients were categorized into four groups based on CEA trajectory, and the analysis showed that the hazard of death was higher in all groups—decreasing (HR = 1.27), early-rising (HR = 4.50), and later-rising (HR = 3.68)—compared to the low-stable group [31].

Serum CEA measurement was reported in one study to have a sensitivity of 83.67% for the diagnosis of liver cancer [32]. Moreover, serum CEA levels were found to be significantly higher in cases of hepatocellular carcinoma (HCC) when the disease was metastatic [33]. The cut-off point for CEA was >5.0 ng/mL, and measurements were performed using a microparticle chemiluminescence assay alongside other markers, such as PIVKA-II (Protein Induced by Vitamin K Absence-II) and AFP [33]. Among these, CEA demonstrated the highest AUC (0.849) for distinguishing metastatic disease from chronic liver disease [33]. Another valuable study compared HCC patients who underwent hepatectomy with healthy controls, measuring preoperative serum levels of CEA, CA19-9, and AFP using radioimmunoassay [34]. Firstly, serum CEA levels in HCC patients (11.18 ± 2.61 ng/mL) were significantly higher than those in healthy controls (1.06 ± 0.13 ng/mL), particularly in cases with distant metastases (13.72 ± 2.88 ng/mL) [34]. Secondly, six months after surgery, serum concentrations of the three markers were higher in patients with recurrent disease (12.62 ± 4.75 ng/mL) compared to those who remained relapse-free (5.22 ± 2.38 ng/mL) [34].

Certain studies have indicated that the role of serum CEA in breast cancer is relatively limited. Therefore, efforts have been made to improve its sensitivity, and specificity by combining it with other biomarkers. A multi-marker panel was developed using ELISA or electrochemiluminescence immunoassay to measure the levels of FTO (fat mass and obesity-associated protein), PIK3CB (phosphatidylinositol-4,5-biphosphate 3-kinase catalytic subunit β), CEA, and CA15-3 in patients with stage I–II breast cancer [35]. As a result, the study confirmed that the inclusion of all four markers leads to superior diagnostic accuracy compared to CEA and CA15-3 alone [35]. One of the publications analyzed 961 breast cancer cases based on preoperative serum concentrations of CEA, CA15-3, and CA125, using a cut-off value of 5 ng/mL for CEA [36]. It was revealed that higher CEA levels correlated with larger tumor size, nodal involvement, and HER2-positive status [36]. Conversely, another study involving 482 female breast cancer patients did not demonstrate any relationship between CEA levels or nodal involvement and observed higher serum CEA concentrations in HER2-negative cases [37].

CEA has found a particularly important application in colorectal cancer, mainly in the surveillance of patients for recurrence, where a concentration of 10 ng/mL has been proposed as a cut-off point, indicating the need to implement an in-depth diagnosis [38]. It is noteworthy that the combination of serum CEA concentrations with a CT scan can lead to an increase in the early detection of metastases and the number of surgical interventions as a treatment modality for these metastases. However, this combination did not affect OS [39,40].

Often, the highest serum CEA levels are observed in patients with colorectal cancer metastases located in the liver. This phenomenon can be attributed, in part, to the fact that the tumor cells responsible for metastatic growth are the primary source of CEA, and the liver’s abundant vascularity facilitates the entry of CEA into the circulation. Furthermore, the liver is the primary site of CEA degradation; therefore, its occupation by metastatic foci is associated with impaired function, including the metabolization of CEA. Elevated serum CEA levels detected prior to surgery in colorectal cancer patients are indicative of more advanced or metastatic cancer. A correlation has been identified between elevated serum CEA levels prior to surgery and a diminished (OS), disease-free survival (DFS), and an elevated risk of mortality among patients with stage I–III colorectal cancer [38]. Postoperative failure of serum CEA concentrations to return to reference values or concentrations >10 ng/mL suggested recurrence or incomplete resection, and postoperative concentrations above 10 ng/mL were an indicator of the presence of secondary foci [38]. Preoperative serum CEA levels have also been shown to be a predictor of a poorer response to neoadjuvant treatment for rectal cancer, which can adversely affect pCR (pathological complete response) and OS [38,41,42].

The serum concentration of CEA, along with other markers, has become part of modern diagnostics through its inclusion in multi-marker panels. For instance, CA19-9 has been found useful in predicting tumor stage, resectability, and prognosis in patients with pancreatic cancer, but the doubts considering limited sensitivity in early-stage cases remain valid. One of the studies reported a rise in CA19-9 serum levels beginning two years before pancreatic cancer diagnosis, with diagnostic performance peaking at 60% sensitivity and 99% specificity during the final 6 months prior to diagnosis [43]. Notably, the inclusion of LRG1 (leucine-rich alpha-2-glycoprotein 1) and TIMP1 (metallopeptidase inhibitor 1) as additional protein markers in cases where CA19-9 alone was insufficient, resulted in a 13.2% increase in sensitivity at 99% specificity [43]. The concentrations of the three mentioned markers were measured using bead-based ELISA assays [43]. Interestingly, the analysis conducted across 19 studies demonstrated that CEA-based panels—combining markers such as CEA, CA19-9, CA125, CA50 (cancer antigen 50), and CA242 (cancer antigen 242) in various configurations—provided greater diagnostic accuracy for pancreatic cancer than CEA or CA19-9 alone [44]. However, this improvement did not translate into higher sensitivity, which remained comparable to that of the individual markers [44]. Another study comparing patients who developed invasive epithelial ovarian cancer with healthy controls suggests that a longitudinal multi-marker panel—including CA125, CA72-4, and HE4 (human epididymis protein 4)—is superior to CA125 alone in the early detection of ovarian cancer [45]. What is worth remembering, in general, approximately 20% of early-stage ovarian cancer cases are not associated with CA125 alterations. Thus, a crucial aspect of this finding is that the addition of HE4 and CA72-4 to the diagnostic panel enabled the identification of 16% (4/25) of screen-negative cases [45]. Interestingly, although CEA was among the biomarkers with the highest initial diagnostic potential, it was not included in the final panel that achieved the greatest sensitivity (83.2%) at 98% specificity [45].

## 3. Cancer—Peritoneal Fluid CEA

As previously stated, cancer cells can be present not only in the bloodstream, but also in other body fluids, including the peritoneal fluid. Such micro-metastases represent a significant component of the process of cancer recurrence, as evidenced by studies conducted on gastric cancer and gynecological cancers [46].

Despite the established role of cytology as the gold standard for the detection of tumor cells in body fluids, its sensitivity remains relatively low. Therefore, determining the probability of recurrence based solely on their presence remains a significant challenge [46]. The sensitivity of cytology in this context ranges from 50% to 70%, given that peritoneal involvement of the tumor does not necessarily equate to the release of tumor cells from this origin [47]. Taking this into consideration, it is important to acknowledge that there are cases of peritoneal recurrence in the absence of changes in peritoneal fluid cytology. A potential approach to enhance the sensitivity of cytology involves the integration of cytological analysis with immunohistochemical testing, using antibody panels [48]. In an effort to assess the prognostic value of peritoneal biomarkers in colorectal cancer surgery, 189 patients were prospectively included from a total of 234 undergoing abdominal procedures [49]. Tumor markers (CEA and CA19-9) and cytology were prospectively evaluated in both peritoneal effusion and peritoneal irrigation fluid [49]. Tumor marker levels were measured using radioimmunoassay [49]. Higher peritoneal fluid concentrations of CEA (>4 ng/mL vs. ≤4 ng/mL) and CA19-9 (>37 ng/mL vs. ≤37 ng/mL) were found to correlate with both poorer cancer-free survival (CFS) and OS [49]. Interestingly, even in cases with negative peritoneal cytology, elevated peritoneal CEA levels remained significantly associated with both recurrence and peritoneal metastatic recurrence [49].

Consequently, there is a significant need to develop a reproducible method for evaluating CEA concentration in peritoneal fluid in the context of oncology. To date, it has been demonstrated that both CEA protein and CEA mRNA testing to predict post-surgical recurrence of gastric cancer exhibited higher sensitivity (although lower specificity) than cytology [50]. The assessment of CEA protein can be performed using radioimmunoassay, immunoassay, or ISTA (sphere turbidimetric assay) techniques, while RT-PCR (reverse-transcriptase polymerase chain reaction), real-time RT-PCR, or TRCR (transcription-reverse transcription concerted reaction) [46] are helpful for measuring CEA mRNA. Despite the indisputable benefit of these tests, which is their significant sensitivity, the possibility of false-positive results cannot be discounted. One potential explanation for this phenomenon is that other cells present in the peritoneal fluid, such as macrophages, leukocytes, or necrotic tumor cells, possess the capacity to secrete CEA, thereby potentially altering the reliability of the study [50].

Few studies have demonstrated the high degree of specificity, sensitivity, and accuracy of markers such as CEA, CA15-3 (cancer antigen 15-3), and CA19-9, with percentages ranging from 86 to 96% in the context of differentiating malignant from benign effusions [15,51]. Interestingly, a diagnostic algorithm has been proposed, which is based on the ascites concentration of tumor markers (CEA, CA15-3, and CA19-9) and their ascites to serum concentration ratio (A/S) [52]. This algorithm has been demonstrated to facilitate the determination of the probable nature of the effusion, whether malignant or benign, with a high degree of accuracy. Patients with a maximum value of A/S CEA, A/S CA15-3, or A/S CA19-9 ≥ 1.5 in the presence of negative ascitic tumor markers, or ≥1 in the presence of positive ascitic tumor markers, were considered to have malignant ascites [52]. In contrast, maximum values < 1 with negative markers and <0.67 with positive markers were indicative of benign ascites [52]. Furthermore, it has been observed to identify a category of patients who require more complex management to ascertain the underlying cause of the effusion. Patients with a maximum value of A/S CEA, A/S CA15-3, or A/S CA19-9 < 1.5 in the presence of negative ascitic tumor markers, and <1 in the presence of positive markers, were classified as having ascites of undetermined origin [52].

Concurrently, another author concluded that the concentration of a single marker in the peritoneal fluid is not sufficiently reliable and only the combination of several markers allows hypotheses to be made about potential peritoneal involvement of the tumor [53]. Moreover, a review paper asserts that, despite the established correlation between elevated CEA levels in the ascites and an increased likelihood of a neoplastic origin, this marker lacks sufficient sensitivity for diagnosis of a cancer-related origin based solely on its levels [3].

Considering the challenges associated with using CEA as a marker in ascitic fluid, the need to integrate additional diagnostic modalities becomes increasingly apparent. The importance of combining diagnostic tools is further highlighted by a study demonstrating that CEA, AFP (α-fetoprotein), and CA19-9 are trustworthy indicators, particularly in conjunction with PET-CT, for differentiating the etiology of an effusion (neoplastic vs. non-neoplastic) [54].

In view of the above aspects, further research is required into the role and interpretation of CEA levels in the ascites so that the place of this marker in the universal diagnosis of oncology patients can be clearly defined.

## 4. Gynecological, Colorectal, and Gastric Cancers—Peritoneal Fluid CEA

However, the knowledge gained so far has allowed some interdependencies to be established for several clinical scenarios. For example, CEA levels measured using the chemiluminescence method in peritoneal fluid collected intraoperatively are found to be significantly higher (36.3 ± 170.9 ng/mL) in patients with gynecological tumors than in the group suffering from benign gynecological pathologies (0.2 ± 0.95 ng/mL) [22]. The same study also indicates that, although peritoneal fluid cytology plays a prognostic role and helps to decide the intensity of planned therapy, (1) its result does not affect the staging of gynecological cancers, and (2) it carries the risk of false-positive results in benign disease and false-negative results at early stages of cancer [22].

Interestingly, among patients with stage IIIC and IV ovarian cancer and patients with stage IIIA and IV peritoneal mesothelioma, even more than 50% of patients with tumor-related ascites had negative cytology [47]. This work suggests that a ratio of tumor markers (including CEA, CA19-9, and CA15-3, assessed by the means of electrochemiluminescence assay) in the effusion relative to their serum concentration has a higher sensitivity (75.7%) than a single measurement of their concentration in the effusion assuming a high cut-off point [47].

One retrospective study also showed that after abdominal puncture and measurement of CEA levels in the peritoneal fluid, a CEA value > 3.89 ng/mL in the ascites was a predictor of peritoneal carcinomatosis in colorectal cancer [55]. Another research focused on detecting CEA transcripts in peritoneal lavage fluid and serum from 39 patients undergoing curative resection for colorectal cancer [56]. Samples collected both pre- and post-operatively were analyzed using the qPCR method [56]. The study demonstrated that over 50% of patients with positive qPCR results had disseminated colorectal cancer cells present in peritoneal lavage fluid, despite the absence of such cells in blood samples, with 71% developing recurrence [56].

To date, there have also been numerous attempts to use CEA levels in the ascites as a prognostic marker and indicator predicting potential recurrence with peritoneal involvement in patients with gastric cancer.

One meta-analysis found that positive molecular testing that targets, among other things, the CEA gene based on peritoneal fluid correlated with shorter OS and PRF (peritoneal recurrence-free survival) in gastric cancer patients [57]. In addition, another paper found CEA protein levels in peritoneal fluid (0.4–210 ng/mL) to be helpful in predicting and confirming peritoneal recurrence in gastric cancer after surgical resection [50]. CEA protein demonstrated a pooled sensitivity of 77% (95% CI: 0.69–0.84) and specificity of 89% (95% CI: 0.86–0.92) in predicting peritoneal recurrence [50].

The association between the recurrence of surgically treated gastric cancer and the presence of CEA in intraoperatively collected peritoneal fluid has also been confirmed by other authors [46]. On the other hand, in the publication in question, PCR testing of CEA mRNA in peritoneal fluid was found to have such a low positive predictive value (32.0%) that attempting to predict future recurrence based on it seems unreliable [46]. At the same time, PCR of CEA mRNA using peritoneal fluid in the aforementioned group of patients showed a 100% negative predictive value, so a negative result from this test may prompt an attempt to achieve a microscopically complete R0 resection [46]. Interestingly, in a group of gastric cancer patients whose peritoneal fluid cytology obtained intraoperatively proved to be negative, cases with CEA mRNA positivity assessed by qPCR were associated with shorter PFS compared to those in which CEA mRNA was not detected [58]. It is worth bearing in mind that the method of detection of CEA in the peritoneal fluid is not without significance. For example, CEA DNA has been found to be more dependable in diagnosing peritoneal involvement in gastric cancer than CEA mRNA, not least because of the former’s greater stability [59].

## 5. Conclusions

CEA has become a widely utilized diagnostic tool in numerous medical fields, including oncology. Even though our current knowledge of CEA is considerable, certain aspects remain insufficiently investigated, making further research highly valuable for both clinicians and patients. Given CEA’s great potential, briefly outlined in this paper, future studies should focus on establishing more universal standards for cut-off points and measurement methods of CEA in accurately defined patient groups. Additionally, exploring CEA concentrations in body fluids other than serum could provide additional valuable insight.

## Data Availability

Source data are available on request.

## Abbreviations

The following abbreviations are used in this manuscript:

AFP	Alpha-fetoprotein
A/S	Ascites to serum concentration ratio
CA125	Cancer antigen 125
CA15-3	Cancer antigen 15-3
CA19-9	Cancer antigen 19-9
CA242	Cancer antigen 242
CA50	Cancer antigen 50
CA72-4	Cancer antigen 72-4
CEA	Carcinoembryonic antigen
CFS	Cancer-free survival time
CT	Computed tomography
DFS	Disease-free survival
ELISA	Enzyme-linked immunosorbent assay
FTO	Fat mass and obesity-associated protein
GPI-PLD	Glycosylphosphatidylinositol-specific phospholipase D
HCC	Hepatocellular carcinoma
HE4	Human epididymis protein 4
ISTA	Sphere turbidimetric assay
LRG1	Leucine-rich alpha-2-glycoprotein 1
mRNA	Messenger ribonucleic acid
NSCLC	Non-small cell lung cancer
OS	Overall survival time
PET-CT	Positron emission tomography-computed tomography
PIK3CB	Phosphatidylinositol-4,5-biphosphate 3-kinase catalytic subunit β
PIVKA-II	Protein induced by vitamin K absence-II
pCR	Pathological complete response
PRF	Peritoneal recurrence-free survival
qPCR	Real-time quantitative polymerase chain reaction
RT-PCR	Reverse-transcriptase polymerase chain reaction
TRCR	Transcription-reverse transcription concerted reaction

## References

[1] Kankanala V.L., Zubair M., Mukkamalla S.K.R. (2025). Carcinoembryonic Antigen. StatPearls.

[2] Adinolfi M., Delves P.J. (1998). Embryonic Antigens. Encyclopedia of Immunology.

[3] Ahadi M., Tehranian S., Memar B., Vossoughinia H., Salari M., Eskandari E., Farzanehfar M., Sadeghi R. (2014). Diagnostic value of carcinoembryonic antigen in malignancy-related ascites: Systematic review and meta-analysis. Acta Gastro-Enterol. Belgica.

[4] Gold P., Shuster J., Freedman S.O. (1978). Carcinoembryonic antigen (CEA) in clinical medicine. Historical perspectives, pitfalls and projections. Cancer.

[5] Thomas P., Zamcheck N. (1983). Role of the liver in clearance and excretion of circulating carcinoembryonic antigen (CEA). Dig. Dis. Sci..

[6] Jessup J.M., Thomas P. (1989). Carcinoembryonic antigen: Function in metastasis by human colorectal carcinoma. Cancer Metastasis Rev..

[7] Morell A.R. (1992). CEA Serum Levels in Non-Neoplastic Disease. Int. J. Biol. Markers.

[8] Naghibalhossaini F., Ebadi P. (2006). Evidence for CEA release from human colon cancer cells by an endogenous GPI-PLD enzyme. Cancer Lett..

[9] Sack T.L., Gum J.R., Low M.G., Kim Y.S. (1988). Release of Carcinoembryonic Antigen from Human Colon Cancer Cells by Phosphatidylinositol-Specific Phospholipase C.

[10] Kinugasa T., Kuroki M., Yamanaka T., Matsuo Y., Oikawa S., Nakazato H., Matsuota Y. (1994). Non-proteolytic release of carcinoembryonic antigen from normal human colonic epithelial cells cultured in collagen gel. Int. J. Cancer.

[11] Thomas P., Hems D.A. (1975). The hepatic clearance of circulating native and asialo carcinoembryonic antigen by the rat. Biochem. Biophys. Res. Commun..

[12] Hao C., Zhang G., Zhang L. (2019). Serum CEA levels in 49 different types of cancer and noncancer diseases. Progress in Molecular Biology and Translational Science.

[13] Tomasevic Z., Jelic S., Nikolic L., Filipovic I., Stamatovic L., Radosavljevic D. (2003). Negative CEA values in Metastatic Colorectal Carcinoma and the Likelihood of Complete Chemotherapy Response. Int. J. Biol. Markers.

[14] Herbeth B., Bagrel A. (1980). A study of factors influencing plasma CEA levels in an unselected population. Oncodev. Biol. Med. J. Int. Soc. Oncodev. Biol. Med..

[15] Faria D.K., Faria C.S., Doi D., Acencio M.M.P., Antonangelo L. (2019). Hybrid panel of biomarkers can be useful in the diagnosis of pleural and peritoneal effusions. Clin. Chim. Acta.

[16] Wu G., Wang D., Xiong F., Wang Q., Liu W., Chen J., Chen Y. (2024). The emerging roles of CEACAM6 in human cancer (Review). Int. J. Oncol..

[17] Bramswig K.H., Poettler M., Unseld M., Wrba F., Uhrin P., Zimmermann W., Zielinski C.C., Prager G.W. (2013). Soluble Carcinoembryonic Antigen Activates Endothelial Cells and Tumor Angiogenesis. Cancer Res..

[18] Kammerer R., von Kleist S. (1996). The carcinoembryonic antigen (CEA) modulates effector-target cell interaction by binding to activated lymphocytes. Int. J. Cancer.

[19] Wirth T., Soeth E., Czubayko F., Juhl H. (2002). Inhibition of endogenous carcinoembryonic antigen (CEA) increases the apoptotic rate of colon cancer cells and inhibits metastatic tumor growth. Clin. Exp. Metastasis.

[20] Minami S., Furui J., Kanematsu T. (2001). Role of carcinoembryonic antigen in the progression of colon cancer cells that express carbohydrate antigen. Cancer Res..

[21] Soeth E., Wirth T., List H.J., Kumbhani S., Petersen A., Neumaier M., Czubayko F., Juhl H. (2001). Controlled ribozyme targeting demonstrates an antiapoptotic effect of carcinoembryonic antigen in HT29 colon cancer cells. Clin. Cancer Res..

[22] Yildirim M., Suren D., Yildiz M., Alikanoglu A.S., Kaya V., Doluoglu S.G., Aydin O., Yilmaz N., Sezer C., Karaca M. (2013). Tumour Markers in Peritoneal Washing Fluid—Contribution to Cytology. Asian Pac. J. Cancer Prev..

[23] Tomita M., Shimizu T., Matsuzaki Y., Hara M., Ayabe T., Onitsuka T. (2005). Prognostic Significance of Carcinoembryonic Antigen Level in Pleural Lavage Fluid for Patients with Lung Adenocarcinoma. Ann. Thorac. Surg..

[24] Saied G.M., El-Metenawy W.H., Elwan M.S., Dessouki N.R. (2007). Urine carcinoembryonic antigen levels are more useful than serum levels for early detection of Bilharzial and non-Bilharzial urinary bladder carcinoma: Observations of 43 Egyptian cases. World J. Surg. Oncol..

[25] Joshi S., Kallappa S., Kumar P., Shukla S., Ghosh R. (2022). Simple diagnosis of cancer by detecting CEA and CYFRA 21-1 in saliva using electronic sensors. Sci. Rep..

[26] Sarandakou A., Phocas I., Botsis D., Rizos D., Trakakis E., Chryssikopoulos A. (1997). Vaginal fluid and serum CEA, CA125 and SCC in normal conditions and in benign and malignant diseases of the genital tract. Acta Oncol. Stockh. Swed..

[27] Asseo P.P., Panidis D.K., Papaloucas A.C. (1986). Carcinoembryonic antigen activity in human seminal plasma. Int. J. Fertil. Steril..

[28] Tuzun Y., Çelik Y., Bayan K., Yilmaz S., Dursun M., Canoruc F. (2009). Correlation of Tumour Markers in Ascitic Fluid and Serum: Are Measurements of Ascitic Tumour Markers a Futile Attempt?. J. Int. Med. Res..

[29] Nicholson B.D., Shinkins B., Pathiraja I., Roberts N.W., James T.J., Mallett S., Perera R., Primrose J.N., Mant D. (2015). Blood CEA levels for detecting recurrent colorectal cancer. Cochrane Database Syst. Rev..

[30] Grunnet M., Sorensen J.B. (2012). Carcinoembryonic antigen (CEA) as tumor marker in lung cancer. Lung Cancer.

[31] Li C., Liu L., You R., Li Y., Pu H., Lei M., Fan B., Lv J., Liu M., Yan G. (2024). Trajectory patterns and cumulative burden of CEA during follow-up with non-small cell lung cancer outcomes: A retrospective longitudinal cohort study. Br. J. Cancer.

[32] Verma N., Vinocha A. (2023). Role of CA 19.9 and CEA in predicting diagnosis in hepatocellular carcinoma. J. Cancer Res. Ther..

[33] Qi F., Zhou A., Yan L., Yuan X., Wang D., Chang R., Zhang Y., Shi F., Han X., Hou J. (2019). The diagnostic value of PIVKA-II, AFP, AFP-L3, CEA, and their combinations in primary and metastatic hepatocellular carcinoma. J. Clin. Lab. Anal..

[34] Huang X., Li J., Wang F., Hao M. (2018). CT combined with tumor markers in the diagnosis and prognosis of hepatocellular carcinoma. J. BUON.

[35] Mi J., Zhang H., Cao W., Yuan C. (2023). FTO, PIK3CB serve as potential markers to complement CEA and CA15-3 for the diagnosis of breast cancer. Medicine.

[36] Zhao W., Li X., Wang W., Chen B., Wang L., Zhang N., Wang Z., Yang Q. (2021). Association of Preoperative Serum Levels of CEA and CA15-3 with Molecular Subtypes of Breast Cancer. Dis. Markers.

[37] Uygur M.M., Gümüş M. (2021). The utility of serum tumor markers CEA and CA 15–3 for breast cancer prognosis and their association with clinicopathological parameters. Cancer Treat. Res. Commun..

[38] Hall C., Clarke L., Pal A., Buchwald P., Eglinton T., Wakeman C., Frizelle F. (2019). A Review of the Role of Carcinoembryonic Antigen in Clinical Practice. Ann. Coloproctol..

[39] Rosati G., Ambrosini G., Barni S., Andreoni B., Corradini G., Luchena G., Daniele B., Gaion F., Oliverio G., Duro M. (2016). A randomized trial of intensive versus minimal surveillance of patients with resected Dukes B2-C colorectal carcinoma. Ann. Oncol..

[40] Primrose J.N., Perera R., Gray A., Rose P., Fuller A., Corkhill A., George S., Mant D., for the FACS Trial Investigators (2014). Effect of 3 to 5 Years of Scheduled CEA and CT Follow-up to Detect Recurrence of Colorectal Cancer: The FACS Randomized Clinical Trial. JAMA.

[41] Zhang Q., Liang J., Chen J., Mei S., Wang Z. (2021). Predictive Factors for Pathologic Complete Response Following Neoadjuvant Chemoradiotherapy for Rectal Cancer. Asian Pac. J. Cancer Prev..

[42] Hu H., Huang J., Lan P., Wang L., Huang M., Wang J., Deng Y. (2018). CEA clearance pattern as a predictor of tumor response to neoadjuvant treatment in rectal cancer: A post-hoc analysis of FOWARC trial. BMC Cancer.

[43] Fahrmann J.F., Schmidt C.M., Mao X., Irajizad E., Loftus M., Zhang J., Patel N., Vykoukal J., Dennison J.B., Long J.P. (2021). Lead-time trajectory of CA19-9 as an anchor marker for pancreatic cancer early detection. Gastroenterology.

[44] Meng Q., Shi S., Liang C., Liang D., Xu W., Ji S., Zhang B., Ni Q., Xu J., Yu X. (2017). Diagnostic and prognostic value of carcinoembryonic antigen in pancreatic cancer: A systematic review and meta-analysis. OncoTargets Ther..

[45] Simmons A.R., Fourkala E.O., Gentry-Maharaj A., Ryan A., Sutton M.N., Baggerly K., Zheng H., Lu K.H., Jacobs I., Skates S. (2019). Complementary longitudinal serum biomarkers to CA125 for early detection of ovarian cancer. Cancer Prev. Res..

[46] Kim Y.-J., Chung W.C., Choi S., Jung Y.D., Lee J., Chae S.Y., Jun K.-H., Chin H.-M. (2017). The Detection of Messenger RNA for Carcinoembryonic Antigen and Cytokeratin 20 in Peritoneal Washing Fluid in Patients with Advanced Gastric Cancer. Korean J. Gastroenterol..

[47] Trapé J., Sant F., Montesinos J., Arnau A., Sala M., Figols C., Franquesa J., Esteve-Valverde E., Pérez R., Aligué J. (2020). Comparative Assessment of Two Strategies for Interpreting Tumor Markers in Ascitic Effusions. In Vivo.

[48] Benevolo M., Mottolese M., Cosimelli M., Tedesco M., Giannarelli D., Vasselli S., Carlini M., Garofalo A., Natali P.G. (1998). Diagnostic and prognostic value of peritoneal immunocytology in gastric cancer. J. Clin. Oncol..

[49] Lee I.K., Kim D.H., Gorden D.L., Lee Y.S., Sung N.Y., Park G.-S., Kim H.J., Kang W.K., Park J.K., Ahn C.H. (2009). Prognostic Value of CEA and CA 19-9 Tumor Markers Combined with Cytology from Peritoneal Fluid in Colorectal Cancer. Ann. Surg. Oncol..

[50] Xiao Y., Zhang J., He X., Ji J., Wang G. (2014). Diagnostic values of carcinoembryonic antigen in predicting peritoneal recurrence after curative resection of gastric cancer: A meta-analysis. Ir. J. Med. Sci..

[51] Du L., Wei X., Xiao Z., Wang H., Song Y. (2022). Utility of ascitic tumor markers and adenosine deaminase for differential diagnosis of tuberculous peritonitis and peritoneal carcinomatosis. BMC Gastroenterol..

[52] Liu M. Diagnostic Algorithm Based on Ratio of Ascites-Serum Tumor Markers is Superior to Tumor Markers in the Differentiation of Benign Ascites from Malignant Ascites—PubMed. https://pubmed.ncbi.nlm.nih.gov/38880300/.

[53] Jain T. (2022). Ascitic and serum levels of tumor biomarkers (CA 72-4, CA 19-9, CEA and CA 125) in discrimination of cause of ascites: A prospective study. Arq. Gastroenterol..

[54] Gan X., Yang J., Wang L., Tan B., Wang L. (2020). Application of PETCT imaging information combined with tumor markers in etiological screening of infectious and non-infectious ascites. J. Infect. Public Health.

[55] Song S.E., Choi P., Kim J.H., Jung K., Kim S.E., Moon W., Park M.I., Park S.J. (2018). Diagnostic Value of Carcinoembryonic Antigen in Ascites for Colorectal Cancer with Peritoneal Carcinomatosis. Korean J. Gastroenterol..

[56] Guller U., Zajac P., Schnider A., Bösch B., Vorburger S., Zuber M., Spagnoli G.C., Oertli D., Maurer R., Metzger U. (2002). Disseminated Single Tumor Cells as Detected by Real-Time Quantitative Polymerase Chain Reaction Represent a Prognostic Factor in Patients Undergoing Surgery for Colorectal Cancer. Ann. Surg..

[57] Deng K., Zhu H., Chen M., Wu J., Hu R., Tang C. (2016). Prognostic Significance of Molecular Analysis of Peritoneal Fluid for Patients with Gastric Cancer: A Meta-Analysis. PLoS ONE.

[58] Nakanishi K., Kanda M., Umeda S., Tanaka C., Kobayashi D., Hayashi M., Yamada S., Kodera Y. (2019). The levels of SYT13 and CEA mRNAs in peritoneal lavages predict the peritoneal recurrence of gastric cancer. Gastric Cancer.

[59] Harada H., Soeno T., Nishizawa N., Washio M., Sakuraya M., Ushiku H., Niihara M., Hosoda K., Kumamoto Y., Naitoh T. (2021). Prospective study to validate the clinical utility of DNA diagnosis of peritoneal fluid cytology test in Gastric cancer. Cancer Sci..

